# Capturing Home Care Information Management and Communication Processes Among Caregivers of Older Adults: Qualitative Study to Inform Technology Design

**DOI:** 10.2196/53289

**Published:** 2024-07-04

**Authors:** Ryan Tennant, Sana Allana, Kate Mercer, Catherine M Burns

**Affiliations:** 1 Department of Systems Design Engineering Faculty of Engineering University of Waterloo Waterloo, ON Canada; 2 Library University of Waterloo Waterloo, ON Canada

**Keywords:** aged, caregivers, patient safety, communication, patient care team, information management, digital technology, human-centered design, mobile phone

## Abstract

**Background:**

The demand for complex home care is increasing with the growing aging population and the ongoing COVID-19 pandemic. Family and hired caregivers play a critical role in providing care for individuals with complex home care needs. However, there are significant gaps in research informing the design of complex home care technologies that consider the experiences of family and hired caregivers collectively.

**Objective:**

The objective of this study was to explore the health documentation and communication experiences of family and hired caregivers to inform the design and adoption of new technologies for complex home care.

**Methods:**

The research involved semistructured interviews with 15 caregivers, including family and hired caregivers, each of whom was caring for an older adult with complex medical needs in their home in Ontario, Canada. Due to COVID-19–related protection measures, the interviews were conducted via Teams (Microsoft Corp). The interview guide was informed by the cognitive work analysis framework, and the interview was conducted using storytelling principles of narrative medicine to enhance knowledge. Inductive thematic analysis was used to code the data and develop themes.

**Results:**

Three main themes were developed. The first theme described how participants were continually *updating the caregiver team*, which captured how health information, including their communication motivations and intentions, was shared among family and hired caregiver participants. The subthemes included *binder-based health documentation*, *digital health documentation*, and *communication practices beyond the binder*. The second theme described how participants were *learning to improve care and decision-making*, which captured how they acted on information from various sources to provide care. The subthemes included *developing expertise as a family caregiver* and *tailoring expertise as a hired caregiver*. The third theme described how participants experienced *conflicts within caregiver teams*, which captured the different struggles arising from, and the causes of, breakdowns in communication and coordination between family and hired caregiver participants. The subthemes included *2-way communication* and *trusting the caregiver team*.

**Conclusions:**

This study highlights the health information communication and coordination challenges and experiences that family and hired caregivers face in complex home care settings for older adults. Given the challenges of this work domain, there is an opportunity for appropriate digital technology design to improve complex home care. When designing complex home care technologies, it will be critical to include the overlapping and disparate perspectives of family and hired caregivers collectively providing home care for older adults with complex needs to support all caregivers in their vital roles.

## Introduction

### Background

The health care landscape is undergoing a significant transformation with the advent of home care services; yet, unlike a regulated hospital or formal care setting, home care environments lack standardization in the design and implementation of technologies that support health information management and communication [1,2]. Effective health information sharing is essential for patient-centered care and shared decision-making to reduce the risk of adverse events [3-5]. Adverse events in the home are commonly associated with insufficient communication and coordination issues among family and hired caregivers [6-8]. In Canada and the United States, the proportion of people receiving home care who experience adverse events ranges from 4.2% to 13% [7,9].

With respect to home care for older adults, their health conditions and health information are often highly varied, their living environments are disparate and often involve diverse caregiver teams, and caregivers often attempt to implement nonelectronic systems to manage recordkeeping and information sharing [1,10,11]. The rapidly expanding digital world has left home care behind [12], which could be attributed to the sociotechnical complexities that make it challenging to design standardized technologies for this care environment [13-17]. These complexities must be addressed to ensure that all caregivers can access the benefits of digitalization and provide better care.

Prior work on the design and integration of digital technology for caregivers primarily focuses on the perspectives of family caregivers [12]. Researchers have examined family caregivers’ general needs for the design of home care technologies [18]; how family caregivers select and use assistive technologies [19,20]; the factors influencing family caregivers’ satisfaction with, or adoption of, technologies [21,22]; and design considerations to help family caregivers manage specific health care conditions [23,24]. Other researchers have more closely examined information management through family caregiver handoff processes in the home [25,26] and described how home care nurses desire a digital dashboard to support evidence-based care provision [17]; it has also been recommended to focus on quality improvement when adopting IT, along with addressing problems with information sharing, reducing excessive documentation, and providing training [15]. While recognizing the involvement of diverse care teams in complex home care, limited qualitative analyses have examined family *and* nonfamilial or hired caregivers [12]. There is also a need to establish a stronger contextual understanding of information management and communication experiences to inform technology design for complex home care environments [12].

### Complex Home Care

People with complex home care needs include diverse individuals who typically experience any combination of chronic conditions, multimorbidity, mental health issues, polypharmacy, and social vulnerability [27-29]. They also face significant barriers to receiving optimized care in their homes [27-29].

A systematic review of home care for older adults in Canada identified that mobility issues, cognitive impairment or mental illness, and chronic conditions are typical characteristics of older adult home care recipients with greater impacts on their quality of life and need for caregiving support at home [2]; for example, older adults with mobility issues may restrict their physical activity due to a fear of falling, especially if they have fallen before, and may receive home-based rehabilitation [2]. Cognitive impairments such as Alzheimer disease and mental health illnesses such as depression or suicidal behaviors are often associated with an increased need for home care workers and family caregiver support [2]. Older adults living with disabilities and chronic illnesses such as hypertension, heart problems, diabetes, and arthritis have very high care needs [2]. Furthermore, among older Indigenous adults, the incidence of these conditions is 2 to 3 times higher than the average rate for older adults in Canada [2].

With an increasingly aging population [30], the proportion of people requiring complex home care is also growing; for example, in Ontario, Canada, it is projected that, by 2040, the number of older adults (aged ≥80 years) will more than double, with 1 in 5 having complex care needs [30]. While the ongoing COVID-19 pandemic adds new challenges with individuals already managing complex home care [31,32], it also contributes to new complex care needs of individuals experiencing postacute sequelae of SARS-CoV-2 infection and other long-term health impacts from reinfection [33-35].

The rise in the number of people needing complex care at home has increased the demand for caregivers. Caregivers of people with long-term home care needs are often unpaid family members who work 20 to 40 hours per week providing care [36]. Approximately 75% of caregivers in Ontario have expressed worries about managing their caregiving duties, and 42.1% report feeling distressed [36,37]. To supplement and support the work of family caregivers, approximately 20 million caregiving visits or home care support services are purchased annually from service provider organizations to help individuals remain in their homes when receiving care [38].

### Study Objectives

While family caregivers are critical, it is important to remember that complex home care often requires communication across caregiver teams, including hired caregivers (in this study, hired caregivers refers to individuals such as personal support workers [PSWs] and home care nurses). Indeed, Wolff et al [39] describe the significant potential for health IT to support stronger partnerships between family caregivers and health care workers. Moreover, Lindeman et al [12] highlight key research needs in addressing the design and use of technology to support health documentation and information exchange among caregivers. There are significant gaps in existing literature informing the design of complex home care technologies that considers family and hired caregiver experiences and the integration of future technologies for health information management and communication [12].

The objectives of this study are twofold: to explore (1) the health documentation experiences of family and hired caregivers and (2) their experiences with health communication with other caregivers in the context of their respective home care environments. The results from this work provide insight into potential users’ personal, physical, and social care environments, which is a critical foundational step in informing the design and adoption of new technologies for complex home care [40,41].

## Methods

### Study Design

This research is part of a larger interview study about the perspectives of caregivers across North America regarding health IT development to support information management and communication in complex home care [42,43]. The first part of this research focused on caregivers’ experiences—1 study focused on family caregivers of children with special health care needs [42], while the results reported in this study focused on family and hired caregivers of older adults. The second part of this larger study explored caregivers’ expectations regarding using voice assistants to interface with health care information in the home [43].

Despite their functional differences and the unique training and education that hired caregivers have, both family and hired caregivers were included in this study because of their complementary roles and the critical need to capture unique perspectives from a holistic approach, including physical, emotional, and social dimensions of home care. We also recognize the expertise of the family caregiver. Therefore, we use the term “caregiver” in this study to encompass family and hired caregivers and have noted when hired caregiver participants were home care nurses or PSWs.

### Ethical Considerations

A research ethics board at the University of Waterloo reviewed and granted ethics clearance for the study (42179). All participants were interviewed virtually from their homes due to COVID-19–related protection measures in place. Per the ethics approval, and given that this study involved minimal risk to participants, informed consent was obtained verbally. Participant data were stored electronically on a password-protected account that only the research team could access, deidentified, and referred to only by a participant code (eg, participant 1). Participants were not remunerated for their participation. Each participant received a thank-you letter after the interview.

### Participants and Data Collection

Participants were eligible if they were aged at least 18 years and either a family caregiver or a hired caregiver for an older adult (aged ≥65 years) who required complex care services in their home anywhere in North America. Complex care refers to the care of individuals who need care services for any combination of chronic conditions, mental health issues, medication-related problems, and social vulnerability. In this study, a family caregiver was one who provided or coordinated care for a family member, a partner, or a friend; they assisted this person with health- or medical-related tasks in their home. A hired caregiver, who was not a family member of the care receiver, was a home care nurse, PSW, or other caregiver employed to provide home care services to an older adult.

Recruitment was supported by disseminating study materials through various nonprofit, for-profit, and public home health care agencies, as well as caregiver support agencies, including Home Care Ontario and other caregiver networks and organizations in Ontario, with which eligible participants may be associated. We also disseminated recruitment materials within groups on social media platforms, including Reddit, Facebook, and Twitter (subsequently rebranded X), that were created for family caregivers, PSWs, and nurses. We followed up with snowball sampling, where participants were asked to share the study poster with other potential participants at the end of their interviews.

One researcher (RT) developed the interview guide, guided by the cognitive work analysis framework and the storytelling principles of narrative medicine to enhance knowledge [44,45], which was reviewed and iterated by 2 researchers (CM and KM; Textbox 1). Two researchers (RT and KM) conducted semistructured interviews with each eligible participant who contacted us about participating in the study. During the interview, the participants were asked about their home care environment, their experiences of providing care, and their coordination experiences with other caregivers. Hired caregivers were asked to speak about their recent work experiences with a client or comment in general terms about their experiences. The interviews were recorded using Microsoft Teams, and only the audio recordings were used for transcription.

**Semistructured interview guide and question prompts for family and hired caregiver participants.**

**Home care environment**

- I’d first like to talk a bit today about the in-home care you have set up or that you work in. Can you tell me about how you’ve navigated caring for them in your home, and what you’ve needed to learn to do to provide care?
- Would you mind speaking more on learning about their condition and the treatments or therapies that they need?
- What different types of technology supports do they need at home?
- Who else is on their care team? What is their role?
- Do they take any medications for their condition? How many medications do they take and how do they take them? How is this information documented?
- How long do you usually provide care for them?

**Caregiving experience**

- Now I’d like to talk more about your experience caring for this person. What are some of the major factors or tensions that influence the quality of care that they receive? You can comment on both before and after COVID-19 if you’ve noticed a change.
- Could you talk a little bit about your feelings of control?
- What information do you need to care for them? What information do you need from others in their caregiver team? What information do you need from other sources?
- How did the setup of their house change to care for them?
- Do you document information about their care in their home? How do you do this? Who can see the information that you record?
- What impacts your ability to document information in their home?
- Have you received formal training to care for them? Have you been trained to use their medical technology? What did the training consist of?

**Coordination with other caregivers**

- You mentioned that there are other caregivers providing care. Are there times when these caregivers take over primary care responsibilities for them? Can you talk a bit about what typically happens when another caregiver takes over primary care responsibilities for them?
- What information do you usually provide to this person?
- How do you communicate this information?
- What would help you communicate information more effectively?
- What tasks does this caregiver do for them?
- Do other caregivers in their health care team record information in their home? What do they record?
- Where do they record this information? Who can see this information?

### Data Analysis

The interview data were stored and organized using NVivo 12 (Lumivero) and Excel 2021 (Microsoft Corp) and analyzed inductively to construct themes [46]. The analysis process involved the following steps: (1) Microsoft Stream’s closed captions feature was used to transcribe the audio recordings; (2) 2 researchers (RT and SA) reviewed and deidentified the transcripts; (3) 2 researchers (RT and SA) listened to the interview recordings and read through the transcripts to familiarize themselves with the data; (4) all interview data were thematically coded using process and open coding to identify patterns and meaning within the transcripts, and the researchers (RT and SA) regularly discussed the codes and their organization [47]; (5) the developed codes from 1 family caregiver transcript and 1 hired caregiver transcript were applied to each interview, with new codes developed as needed; and (6) the codes were grouped to construct themes that were reviewed and refined by the entire research team. Disagreements were first resolved through discussion between RT and SA; otherwise, another research member (KM) was involved. Interviews were continued until code saturation was reached within the entire data set when no new codes were identified [48].

### Reflexivity Statement

The research team has prior experience providing care for older adults, which may have introduced a bias during the study. These potential effects were mitigated by using 2 researchers (RT and KM) to conduct the interviews, using 2 researchers (RT and SA) during the coding and theme-forming process to challenge any emerging preconceptions and support the reliability of the analysis, and seeking feedback on the preliminary findings from the entire research team. After each interview, the researchers (RT and KM) also conducted a debriefing session to discuss the content, reflect on the participants’ experiences, and make notes.

## Results

### Participant Characteristics

The participants were aged 24 to 83 years, and they cared for various older adult clients and family members (Table 1). Most of the family caregivers (6/9, 67%) provided care to a spouse, while others cared for parents (1/9, 11%), grandparents (1/9, 11%), and siblings (1/9, 11%). Of the 6 hired caregiver participants, 4 (67%) were PSWs, and 2 (33%) were home care nurses. The participants provided home care for durations ranging from 4 months to 13 years. The average time spent providing care across all participants was approximately 5 (SD 3.7) years. Participants generally described caregiving activities for individuals and loved ones with mobility issues, cognitive needs, illnesses, and wound care needs.

**Table 1:** Reported age, gender, and caregiving experiences of family and hired caregiver interview participants (N=15).

Characteristics			Family caregivers (n=9), n (%)		Hired caregivers (n=6), n (%)
**Age (y)**					
	18-24	1 (11)		0 (0)	
	25-34	1 (11)		1 (17)	
	35-44	0 (0)		2 (33)	
	45-54	0 (0)		1 (17)	
	55-64	1 (11)		1 (17)	
	65-74	2 (22)		1 (17)	
	75-84	4 (44)		0 (0)	
**Gender identity**					
	Woman	8 (89)		5 (83)	
	Man	1 (11)		1 (17)	
**Caregiving experience (y)**					
	0-5	6 (67)		4 (67)	
	6-10	2 (22)		1 (17)	
	11-15	1 (11)		1 (17)	
**Caregiving relationship**					
	Client	0 (0)		6 (100)	
	Spouse	6 (67)		0 (0)	
	Parent	1 (11)		0 (0)	
	Grandparent	1 (11)		0 (0)	
	Sibling	1 (11)		0 (0)	

**Figure 1:**
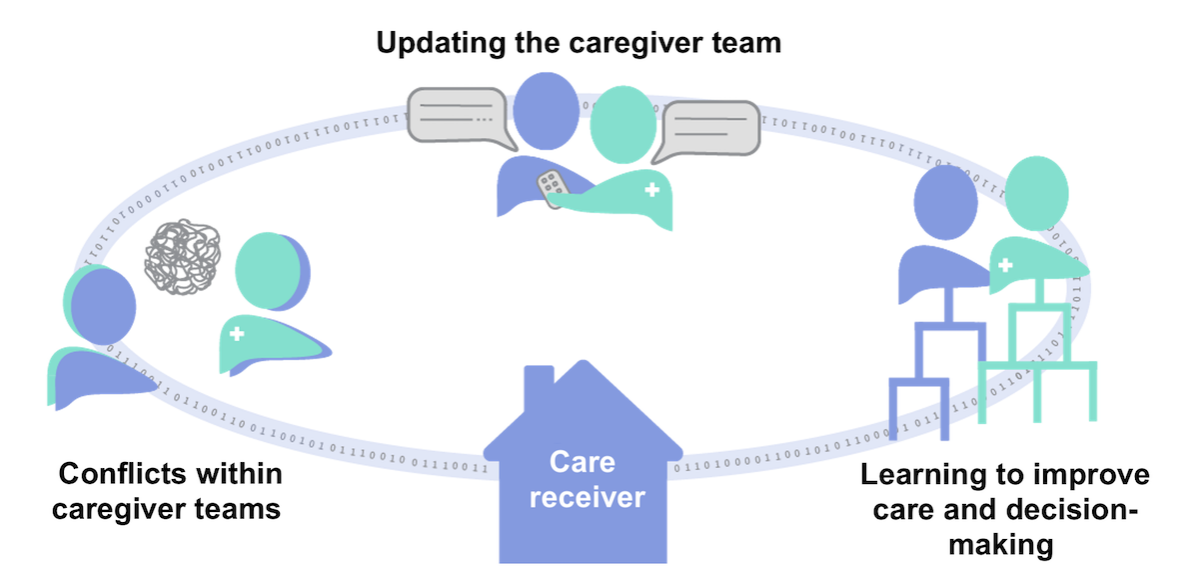
A visual representation of the developed overarching themes that describe family and hired caregiver participants’ experiences with health information management and communication when caring for an older adult, showing how the themes are connected by information flow through the care receiver’s home.

The codes were arranged into 3 main themes that describe the nuanced factors involving technologies, interactions, and tasks essential for health information management and communication in the complex home care environment for older adults (Figure 1; Table 2): (1) updating the caregiver team (binder-based health documentation, digital health documentation, and communication practices beyond the binder), (2) learning to improve care and decision-making (developing expertise as a family caregiver and tailoring expertise as a hired caregiver), and (3) conflicts within caregiver teams (2-way communication and trusting the caregiver team).

**Table 2:** Overarching themes and the list of corresponding codes developed to describe family and hired caregivers’ health information management and communication experiences in complex home care settings for older adults through inductive qualitative analysis.

Themes and subthemes			Codes		
			Family caregivers		Hired caregivers
**Updating the caregiver team**					
	**Binder-based health documentation**				
		Physical documentation Keeping personal notes to support safety Transparency of recordkeeping Managing medications		The burden of physical documentation Keeping personal notes to support safety Capturing holistic information	
	**Digital health documentation**				
		Desire to ease documentation		Concerns regarding information silos	
	**Communication practices beyond the binder**				
		Calling and texting caregivers Ensuring awareness of the patient status Leaving notes for caregivers Reminding caregivers Bringing information to clinicians		Calling and texting caregivers Handing off care Leaving notes around the home	
**Learning to improve care and decision-making**					
	**Developing expertise as a family caregiver**				
		Understanding medications and the health condition Learning from caregivers and health care professionals		—^a^	
	**Tailoring expertise as a hired caregiver**				
		—		Learning from caregivers Having prior knowledge Learning through observation Learning from the client Reviewing documentation and health care records	
**Conflicts within caregiver teams**					
	**2-way communication**				
		Expressing concerns Struggling to share information Sharing the client’s perspective Providing instructions Communicating health concerns with health care professionals Planning together COVID-19 pandemic impacting care continuity		Coordinating with caregivers Establishing communication boundaries Feelings of control over information sharing	
	**Trusting the caregiver team**				
		Talking through tasks Worrying about the quality of care Watching caregivers		—	

^a^Not applicable.

Many of the codes overlapped between family and hired caregivers within the overarching themes of updating the caregiver team and learning to improve care and decision-making (Table 2). There was a reliance on binder-based health documentation, which was a physical burden for some, and there was a desire for digitalizing home care information for everyone involved, while highlighting the importance of capturing holistic care information. Family and hired caregiver participants developed communication and documentation practices beyond the binder by keeping personal notes to support safer home care, managing information exchange with other caregivers via calling and texting, leaving colocated notes as reminders, and handing off information to other caregivers. They also learned from each other but had different learning needs. Their experiences diverged more prominently in their conflicts regarding information communication, coordination, and control stemming from a lack of 2-way communication, the impacts of the COVID-19 pandemic, and trust issues. The experiences relating to the similarities and differences between family and hired caregiver roles are further explained in the following subsections.

### Updating the Caregiver Team

This theme captured the ways in which health information was shared between family and hired caregivers involved in the home, including their communication motivations and intentions. While all participants in this study discussed updating the caregiver team with pertinent health information about an older adult’s care, their methods and reasons for communicating information varied, depending on the context of their home care situation and their caregiving role as a family or hired caregiver.

#### Binder-Based Health Documentation

Overall, every participant in this study described creating written notes maintained in a central location in the home; for example, a family caregiver participant documented medications and recorded details about their spouse’s reactions:

[The hired caregivers] made written notes to all the people in their company that were coming to see [my spouse]...They would have written notes that they kept on top of the refrigerator...I would keep some notes, and there were times when I would make detailed notes about [my spouse’s] reaction to the medication...I had times when I would write things down every day.

Participant 10, family caregiver

While hired caregivers may have more structured in-home documentation, recordkeeping by family caregiver participants varied, depending on the need for tracking information. A hired caregiver participant strongly expressed the need for caregivers of older adults to maintain paper-based records in their homes:

In the home, it’s still very basic now, as much as you can roll your eyes with that...We find it’s also helpful because if every agency has their own electronic information, that’s great for keeping their records, but remember, there’s all these different people coming into the home. Sometimes you need an old-fashioned 3-ring binder to keep everybody straight.

Participant 5, hired caregiver (home care nurse)

Paper records were seen as important for their transparency of documentation and for providing the ability to have information stored in a single location for the family caregiver, or any other caregiver, to review. However, while documenting health information was important in our participants’ complex home care environments, there was a lack of tools to support documentation; for example, family caregiver participants sometimes designed their own detailed recordkeeping forms to organize health information that others in the caregiver team were required to use for documentation and communication during caregiver handoffs:

I just do a nice log sheet and [my hired caregivers] write down if [they] take [their] meds, did you have a bowel movement, [are they] sleeping or not? I’m pretty good at creating a form. They have to fill this in, and that’s how we communicate. One person comes, 1 person leaves, and they just look at the notes.

Participant 8, family caregiver

A hired caregiver participant expressed that they were required to record all details, such as the ones described by participant 8, during their shifts at specific time intervals. This volume of needed paper-based documentation may be one of the most time-demanding aspects for hired caregiver participants to balance with providing physical care tasks. A hired caregiver participant commented on the compounding nature of this burden with the number of clients in their care:

If you work with 10 people, you have to care for them, and you have to document whatever happens to these 10 people. That is why the PSW job is so hard.

Participant 9, hired caregiver (PSW)

The context for which caregivers documented information varied, depending on the severity of the patient’s conditions, such as monitoring for the effects of a new medication, and the caregiver’s recordkeeping motivations. Family caregivers expressed that they were only documenting information when they felt it was necessary to share progress updates or noticeable patterns with health care professionals. Hired caregiver participants strongly believed that updating others in the caregiver team about the health of their clients, including not only their vital signs but also a holistic picture of the patient, was a critically important factor:

But what about that person? What about how they’re feeling that day? What they’re thinking that day? I get tired of reading documents that say, “changed the sheets, toileted them twice...” But how about asking them, “How do you really feel today? I don’t want to hear ‘good.’ I want to know how you really feel. What are your thoughts?” Like, really get into it and document that. None of this “oh, every day, same document” big deal. What’s the point of even documenting?

Participant 4, hired caregiver (PSW)

The holistic information they captured may be less structured than objective measures about a client in their home, which may be more challenging to share but essential for providing quality care.

#### Digital Health Documentation

In some complex home care situations, digital methods were used to share information with other caregivers. When recordkeeping was completed and transferred digitally, this information was used to update health care professionals about changes or updates regarding the patient’s care. Other caregivers in a supervisory role on the team used this information to monitor the events during another caregiver’s shift. A hired caregiver participant who was also a nurse described a web-based system that they used to communicate with PSWs in the home:

[The online system is] between the person who’s in the home as the PSW and the delegating nurse. I can go in to see that information through our system. There’s an additional link where I can log in and see how their night was.

Participant 5, hired caregiver (home care nurse)

The family caregiver participants in this study did not have access to, or know how to use, any potential technologies to see these details other than texting or leaving a voicemail. Systems such as the one used by participant 5 may provide opportunities for family caregivers to see health information updates without physically being in the home. Despite the lack of access, family caregiver participants who controlled the home care recordkeeping were interested in developing digital documentation methods for their caregiver team but did not know how to accomplish this:

I would like to be able to make that easier for [other caregivers]. I don’t know how, but I understand that in some institutions, they do the recordkeeping on computers.

Participant 8, family caregiver

#### Communication Practices Beyond the Binder

With limited access to technologies that can support communication to update caregivers, there was creativity beyond using physical notes kept in 1 area of the home. Caregivers sometimes implemented more prominent written notes and posted them around their client’s houses. The posted messages aimed to provide context-specific information in the locations where actions needed to be taken by other caregivers and as a salient reminder:

We posted notes all over the place. It was the only way! I put them on the bathroom wall for when [the PSWs] came in. There was one for the morning, one for the daytime, one for the evening, and it was simply, “This is what [the client] requires.” It was listed. They didn’t have to search through charts...I had so many thank-yous from PSWs that were coming in.

Participant 13, hired caregiver (home care nurse)

These notes, which were participants 13’s highly effective method for ensuring that other caregivers could see information at the time and place that it was needed, obviated the need for calling or texting and captured the attention of the caregivers when they were providing specific care tasks. However, beyond physical documentation, the other process involved in updating the participant’s caregiver teams was verbal communication during client handoffs. The participants updated others in the caregiver team on new information to ensure their awareness about changes in their home care situation since the incoming caregiver’s last visit. A hired caregiver participant mentioned the details about which they needed to update other caregivers:

Whenever there’s someone’s turn to take over my shift, I would just say that “[They have] been okay. [They have] been very calm, but there are times that [they were] a bit manic.” Usually, I tell them that [they] already ate, that [they] already took [their] meds at this time, and I usually tell [them] that the only thing that’s missing is [their] meds for this hour.

Participant 2, hired caregiver (PSW)

Information shared verbally supplemented the written record by providing a holistic picture of the situation and supported emphasizing time-sensitive details. Some family caregiver participants felt burdened by having to continually communicate with other caregivers about critical safety information that could have severe consequences if not applied correctly. A family caregiver mentioned their concerns with having to update new caregivers in their home on details about keeping their parent safe:

It’s reminding them stuff like [thickening their drinking water], which is a really, really big risk because my [parent] is prone to something called aspiration, which means if [they] eat any food that can go in [their] lungs, which has happened before, then that can develop into pneumonia...We’ve had to take [them] to the hospital multiple times for that, and that can be really scary because someone like [my parent], who is more vulnerable and prone to getting disease and infection. Especially, taking [them] to the hospital like now [during the COVID-19 pandemic] is pretty scary.

Participant 6, family caregiver

There is a potential fear of future adverse events occurring because family caregivers understand the specific risks associated with their home environment. However, when in-person communication was not possible, but essential information needed to be shared with the caregiver team, the participants used telecommunication devices to provide updates via a telephone call or an SMS text message. A family caregiver participant mentioned that they would call their agency if an adverse event occurred in their home:

If it’s really important, then I’ll call the agency and tell them that [their] workers need to know that such and such is happening...like if there’s been a fall, for instance.

Participant 7, family caregiver

There may be an expectation that information communicated to caregiver agencies over the telephone is subsequently shared with other caregivers involved with the client to ensure widespread awareness when visiting the home.

Telecommunication devices may also afford hired caregiver participants the means to have direct communication with caregivers. A hired caregiver participant highlighted the efficiency of this method of sharing information:

Especially with younger people, with younger family members, they will often text me on my work phone. That’s the most efficient way I find, I text. I call, but I find it even easier to text a lot with the visiting nurses who I talked to recently.

Participant 5, hired caregiver (home care nurse)

However, the demographics of the caregivers, the urgency of the information that needs to be shared, time constraints, and the ease of use may be contributing factors to whether telephone calls or SMS text messages can be used as a reliable communication channel for complex home care.

### Learning to Improve Care and Decision-Making

This theme captured how caregivers acted on information from various sources to provide care. Family and hired caregivers in this study continually learned about their patient’s conditions and the nuances of the home care situation to improve the quality of the care they provided and support their decision-making.

#### Developing Expertise as a Family Caregiver

The degree to which family caregivers felt the need to learn new information and develop caregiving expertise resulted from their loved one’s conditions or symptoms, as a family caregiver participant explained:

[My spouse] had delirium frequently, and [I was] trying to navigate through the delirium where you can’t deny what somebody is experiencing in a delirious state...I could never quite understand it.

Participant 10, family caregiver

Although they did not have a medical background, there was a desire among family caregiver participants to better understand what their loved one was experiencing, despite the challenges of overcoming this knowledge gap. Navigating information often felt similar to doing their own research through reading about the condition or symptoms, learning about medical treatments, and gathering information from health care professionals:

I’ve learned that the more you can engage [individuals with Parkinson disease] intellectually and emotionally with contact, with people, and with things that they like and love, the better they are, even with their mobility. I read up on things. I learned about [my sibling’s] medications, and I know the effects of all of them, and I know the effects of that horrible [medication they were] taking that caused psychosis. I’ve got an informational sheet from some of the people who worked with us who have gone on to become RPNs [registered practical nurses] and so on. [They] gave me a whole handout on how to deal with delusional behavior, and I’ve read about it too.

Participant 8, family caregiver

While some information that family caregivers were learning from health care professionals supported their loved ones through improved care, learning more about providing care in the home also supported their well-being, specifically for performing physical tasks. The family caregiver participant further described how they learned to help their sibling’s mobility while also supporting their own health:

I was doing things wrong for a while too. [My sibling has] mobility issues, and [they] would have difficulty getting up out of a chair. We devised a way of counting and using momentum to pull [them] up. Then I realized I’m hurting my back this way. I learned from some of the various physiotherapists and occupational therapists, and they gave us instructions.

Participant 8, family caregiver

It is important to note that the family caregiver participants in this study were not medically trained professionals. Unlike hired caregivers, the family caregiver participants did not have a standardized knowledge base to support medical decision-making or information gathering.

#### Tailoring Expertise as a Hired Caregiver

The health care workers in this study highlighted the importance of their training and background in providing care; for example, a participant noted the importance of their education in recognizing a severe medical issue that could have quickly developed into sepsis, a situation in which a family caregiver might not have responded as promptly:

Well, I was doing it with the knowledge base—the preidentified wounds on [their] leg, ulcers. I knew right away, but someone that didn’t have that background wouldn’t have pushed the issue.

Participant 13, hired caregiver (home care nurse)

A knowledge base helped participant 13 with their perception-action response to the medical issue. However, while family caregiver participants provide a significant amount of care, there may be barriers to developing perception-action responses for those without a medical background.

The health care workers in this study tailored their caregiving expertise through information acquired within the home care environment as well as information provided by family caregivers or clients. Sometimes, members of the at-home caregiver team verbally communicated this information, supporting hired caregivers who were new to the environment, to ensure that the client’s unique preferences were met, as explained by a hired caregiver participant:

I ask the ones who are already in [client 1]’s team, “What does [client 1] want to do? Whenever [client 1’s sibling] is here, I usually ask [them] what things would let [client 1] ease up [their] feelings of uneasiness or what will be their preference? [Or] you ask [the client], “You want me to do it this way or that way?” We have to be keen and diligent when it comes to [their] liking.

Participant 2, hired caregiver (PSW)

Family caregiver participants’ importance in maintaining detailed health records was an approach to supporting all caregivers with a baseline knowledge base and enhancing their capacity to recognize potential medical issues and optimize their caregiving. Learning from physical documentation was necessary to support decision-making for hired caregiver participants who visited multiple clients daily. A hired caregiver explained how they relied on physical notes—documents that included information from the family caregiver and other hired caregivers—to learn about the most recent events that had occurred in the home and make decisions about the safest time for their client to take their medications:

I also look up their records of what happened all throughout the weekend. It’s usually placed on the table here in [my client’s] home. It’s just the first thing that you go over when you come here...You try to summarize what happened and what time [their] previous extra dose was given so that you can say, “OK, we can give [them] an extra dose at this time,” it’s safe to give [them] an extra dose.

Participant 2, hired caregiver (PSW)

However, there remains a cognitive challenge in tailoring caregiving expertise to each home care environment to inform decisions: hired caregivers need to transform information into short summaries to support other in-home caregivers. The time required to transform the information from paper-based records may constrain busy work schedules in complex home care.

### Conflicts Within Caregiver Teams

This theme captured the struggles and breakdowns in communication and coordination among caregivers, which often impacted care continuity and increased their frustration and lack of trust in each other. These conflicts stemmed from unclear roles and responsibilities as well as communication and coordination issues.

#### 2-Way Communication

Communication challenges existed between family caregiver participants and the hired caregivers as well as between hired caregivers, their clients, and other health care professionals. Conflicts were especially evident when there was a barrier to using technologies meant to ensure that 2-way communication was occurring. This was important in situations where caregivers were required to maintain the older adult’s safety in the home. The technologies used to support communication often only provided a 1-way channel, with no feedback or confirmation of the receiving caregivers’ understanding. A hired caregiver stated as follows:

Most of the time, my frustration was with communicating with the home care and caregivers...There was no connection with me. I got to call a number and leave a voice message. I may or may not have heard back.

Participant 13, hired caregiver (home care nurse)

Limitations in communication technologies may result in uncertainties about receiving and promptly understanding care messages. Reliable communication is critical to reduce tensions, given the number of individuals whom caregivers provide care. The challenges identified in communication among caregivers were also evident with hired and family caregivers, where conflicts emerged due to the hierarchies in caregiver teams. Perceived hierarchy issues raised frustrations for a hired caregiver, who was concerned with the effectiveness of the communication (nonstandardized information-sharing methods created stress for the caregiver team and hindered the coordination of information sharing about home care):

I was frustrated in the fact that if I identified a problem, then there needed to be only 1 person calling the doctor’s office, only 1 person calling the [agency]. They didn’t need multiple phone calls from multiple members or care providers because it was not effective. [The family caregiver] had verbally given all of these people consent for me to handle everything [but] then [they] would start calling.

Participant 13, hired caregiver (home care nurse)

The impact of the COVID-19 pandemic meant that hired caregivers were visiting clients less frequently or performing more virtual visits, depending on the severity of health needs:

The visiting frequency really depends on their acuity now and I can see that because of COVID-19...the nursing agencies have pulled that back. Even they went to more virtual visits, which was a huge headspace change for visiting nursing agencies.

Participant 5, hired caregiver (home care nurse)

Shifting to virtual visits would mean that physical binder-based health documents and other caregiving notes would not be readily accessible to hired caregivers. Family caregiver participants discussed the challenge of ensuring that every individual caregiver understood the nuances and preferences within their home care situation at the beginning of the COVID-19 pandemic and their responsibility to find ways to continue ensuring effective communication of their family’s needs regarding home care services:

And we’ve had some trouble with navigating that sort of thing where finding PSWs, especially at a time like now [during the COVID-19 pandemic], is pretty limited. It’s just been a little bit difficult to get them to understand our perspective and what the client needs. What my [parent] needs.

Participant 6, family caregiver

The risk of losing a hired caregiver at the beginning of the COVID-19 pandemic, despite the communication challenges involved in having a hired caregiver care for their loved one, created more stress for family caregivers.

#### Trusting the Caregiver Team

As a result of conflicts over ensuring that specific care needs were being met, some family caregivers felt additional responsibility to monitor the care tasks in their homes, potentially due to a lack of trust. There was an observed need to provide feedback in real time that was specific to their home, which was not always appreciated by the hired caregiver; for example, family caregivers may have lacked trust in the ability of hired caregivers to provide safe care because they did not have expert knowledge about fall risks in their specific home environment:

If I see something not right when I’m with [them] for the last half hour [of their shift], then I will say, “This is not right. You have to stand here, or [my spouse will] fall over.” That kind of thing. Some of them like it, and some of them don’t like it.

Participant 7, family caregiver

The conflict in this context of information sharing may stem from a lack of trust in hired caregivers performing care in their home where they are not familiar with the nuances of the physical space, but the unclear power dynamic regarding who holds primary health information or acts as the lead caregiver may also play a role.

The family caregiver participant also expressed uncertainty about whether the care needs that they had communicated were being met in their absence:

I’m there for half of the shift because [my spouse] does the last half as an exercise plan, and that’s done downstairs. I see it. If there’s a problem, they’ll tell me. But the thing is, I don’t know whether they’re [watching for fall risks] when I’m not around. That’s my biggest worry. I can’t be all there all the time. It’s just not possible.

Participant 7, family caregiver

Ultimately, the uncertainty around hired caregivers’ vigilance with regard to specific safety risks in their homes created anxieties for family caregivers, reducing respite care’s benefits due to a potential lack of trust in others in the caregiver team.

## Discussion

### Principal Findings

This study captured the health documentation and communication experiences of family and hired caregivers in complex home care settings. Most of the participants (13/15, 87%) in this study were women, who often experience higher caregiving burdens and stress [49], aligning with the Canadian literature and 2022 Statistics Canada data that found women to be more likely to be care providers in the home than men [2,50]. The results also identify the overlap in caregiving experiences among the participants regarding how they were updating the caregiver team with new information about the person receiving care and learning to improve care and decision-making through information obtained from caregivers and by other means. In addition, the results identify disparate experiences with respect to conflicts within caregiver teams when communicating health information and coordinating care responsibilities. The insights gained from this study can inform design requirements for technologies that can meet family and hired caregiver needs with respect to supporting team-based caregiving, considering the sociotechnical complexities of this work domain. Understanding caregivers’ experiences within this complex domain is essential to launch such technology development effectively [51].

Although the caregiving situations discussed in this study were complex, the participants described overlapping experiences within the first and second overarching themes and disparate experiences in the third. Technologies designed to keep caregiver teams updated and support learning and decision-making, while alleviating potential communication conflicts, could help both family *and* hired caregivers. A growing body of research describes the importance of including family caregivers as collaborators for home care and bridging their contributions to home care with hired caregivers [13,25,52]. Much of the current literature on home care technologies focuses on either family caregivers [18-26] or home care workers [15-17] when examining design and development. Our study builds on this work by highlighting the potential overlap in design needs and user requirements between these caregiver roles.

First, hired caregivers are often required to manually document their care delivery for the agencies that employ them. As we found in our study, some hired caregivers have access to electronic documentation systems that are not accessible to family caregivers, or they use paper-based systems to communicate information to other caregivers. Family caregivers may develop their own paper-based recordkeeping systems that they ask their caregivers to fill out, which can create a documentation burden for others in the caregiver team. Despite the need to involve family caregivers in digital technology design and the growing literature on family caregivers’ technology needs and experiences [18,19,22,23,25], caregiving documentation research focuses on hired caregivers [17,53-55], likely due to organizational and institutional requirements. Our study identifies that health information generated by family caregivers is important for safety in a home care environment, and future technologies should find accessible ways to include all caregivers to effectively communicate and share documented health information.

Second, given the communication challenges that caregivers described in this study, where family caregivers were concerned with ensuring that hired caregivers understood the nuances of how to safely care for their loved one in the context of their home, both desired to learn from caregivers and other health information. There is a need to combine the findings from the existing body of literature to build technologies that can address these systems-level needs and support coordinated care; for example, to support handoffs, potential technologies may provide value by efficiently gathering health information from users and intelligently transforming it into situational summaries through the use of large language models [56]. As it is vital to recognize family caregivers as important individuals in documenting the care of their loved one [57] and alleviate the high burdens that family and hired caregivers often face [58,59], automating this aspect of health information and communication could provide significant benefits to all caregivers.

Third, by examining the perspectives of family and hired caregivers and how their work intersects, our study identifies some of the ways in which established literature highlighting only a single caregiving perspective can be enhanced to support caregiver teams; for example, within our third theme regarding communication conflicts, we found examples of the lack of fundamental 2-way communication between caregivers, resulting in uncertainty about whether other caregivers received and understood the information shared, along with challenges related to understanding caregivers’ communication roles and responsibilities. Other research on family caregivers recommends that digital systems include secure messaging, customization, shared calendars, checklists, medication lists, and knowledge about the patient’s condition [25]. Our study highlights the importance of considering the work experience perspectives of family and hired caregivers working together. This suggests that technology design should include features that allow caregivers to confirm whether others in the caregiver team have understood shared health information across functionalities to reduce uncertainties in their caregiving tasks; in addition, the ability to share nuanced home environment details could foster trust within the caregiver team.

Other family caregiver–centered research recommends that IT should put the family caregiver in control [24]. While this is critical for situations led by a family caregiver, when multiple types of caregivers are involved, these technologies should consider all caregiving users’ needs [18], such as the needs of hired caregivers. Hired caregivers have a need for control over health documentation processes—likely due to their training, experiences, and their home care agency’s needs—and for setting appropriate communication boundaries with family caregivers because they often provide care for multiple people. Technology design could play an important role in hired caregivers’ relationships with family caregivers and perceived levels of control. Going forward, a design recommendation may include integrating caregiver profiles to support formalizing roles and care coordination responsibilities in the home.

As also indicated in prior work [17,18,23], there is agreement on the need to digitalize health information management and communication processes to support family and hired caregivers; yet, there are no standardized systems that support all caregivers. One of the challenges of building technologies for older adults’ home care environments identified across this study may be the reliance on paper-based records by home health care systems [24], where issues with the ease of use and ease of integration may prevent adoption over the status quo [21]. It is important to highlight that the participants in this study described how paper-based records supported health information documentation and provided an acceptable and effective method for sharing information with other caregivers. Unfortunately, paper-based records inherently lack functionality for real-time 2-way communication; may not support caregivers adapting to change in a fast-paced, dynamic home environment; and may be limited in supporting cognitive work demands across homes due to nonstandard designs [60-62].

As complex home care continues to evolve and family caregivers take on increasingly critical roles and responsibilities that require quick access to information and clearer communication, paper-based communication tools may not be a sufficient information management strategy [63] or a resilient technology during a pandemic. Existing research shows that digital personal health records for home care are perceived as useful in replacing a paper-based system because they keep relevant information in a single location, save physical space [24,25,64], and support information access on ubiquitous technologies such as smartphones and PCs [21]. The successful implementation of new technologies for caregivers of older adults, which would enable them to share information with others in the caregiver team without a physical documentation burden, review health information documented by others in the caregiver team to stay updated on the status of care, and reduce conflicts resulting from poor communication tools, hinges on maximizing the ease of use and satisfaction [21,53,65,66] and minimizing implementation burdens. More research is needed to identify technology designs and implementation strategies for complex home care that address these overarching needs to support the efficacy of care tasks, caregiver engagement, and system-level adoption among all caregivers.

### Strengths and Limitations

To the best of our knowledge, this is one of the first studies that combines the perspectives of family and hired caregivers and their experiences regarding health information management and communication in the context of complex home care during the ongoing COVID-19 pandemic. The interview data captured rich details about the participants’ experiences and how they work with others in the caregiver team, providing insight that can guide the future development of digital technologies that support caregiving of older adults. Our findings support the need for future research to combine these work experience perspectives when developing design requirements based on user needs for complex home care.

The limitations of our study include the concentrated sample of participants from Ontario; the results may not apply to other Canadian provinces and territories or other countries. In addition, the majority of the participants (13/15, 87%) were women, and the sample size for the hired caregivers was limited. We also did not explicitly ask participants about their client’s or loved one’s specific conditions, diseases, or syndromes; however, some participants shared this information, which we have included in the Results section. One participant’s interview transcript data were unavailable for coding or presentation as anonymous quotes because they did not grant permission for their interview to be audio recorded; hence, the data were captured in detailed written notes for the analysis. While we reached code saturation in our analysis, future studies could expand on this work by recruiting caregiver team focus groups, which could contribute further insights into care coordination and communication in this complex care domain.

### Conclusions

This study highlights the overlapping experiences of family and hired caregivers and the challenges they face when communicating health information and coordinating care responsibilities in complex home care settings. The results suggest the need for digitalized solutions that better support caregiver coordination and ease information sharing to consider how design requirements and user needs from 1 caregiving role overlap with those of other caregivers while addressing disparate communication challenges. Going forward, future research should involve the experiences of family and hired caregivers working together in the design and development of such technologies. By addressing these challenges and leveraging the insights from this study, we can improve the quality of care provided to those who need it most and support caregivers in their vital roles.

## Abbreviations

PSW: personal support worker

## Data Availability

The deidentified data sets generated and analyzed during this study are available from the corresponding author on reasonable request.
